# Ensartinib in a primary pulmonary *ALK*-rearranged mesenchymal neoplasm harboring a novel *HMBOX1::ALK* fusion: a case report and literature review

**DOI:** 10.3389/fmed.2025.1572632

**Published:** 2025-05-20

**Authors:** Qi Liang, Xingyu Jiang, Ziwei Zhou, Yali Jiang, Siqi Ni, Tingyu Zhao, Xiao Zhang, Huanhuan Xu, Qixing Gong, Lingxiang Liu

**Affiliations:** ^1^Department of Oncology, The First Affiliated Hospital with Nanjing Medical University, Nanjing, China; ^2^Department of Nuclear Medicine, The First Affiliated Hospital with Nanjing Medical University, Nanjing, China; ^3^Central Laboratory, The Friendship Hospital of Ili Kazakh Autonomous Prefecture, Ili & Jiangsu Joint Institute of Health, Yining, China; ^4^Department of Hematology and Oncology, Department of Geriatric Lung Cancer Research Laboratory, Jiangsu Province Geriatric Hospital, Nanjing, China; ^5^Department of Pathology, The First Affiliated Hospital with Nanjing Medical University, Nanjing, China

**Keywords:** anaplastic lymphoma kinase (*ALK*), homeobox containing 1 (*HMBOX1*), *ALK*-rearranged mesenchymal neoplasm, ensartinib, case report

## Abstract

Anaplastic lymphoma kinase (*ALK*) gene rearrangements have been increasingly detected in mesenchymal neoplasms. *ALK*-rearranged mesenchymal neoplasms occur mainly in superficial tissues but rarely in internal organs. Herein, we firstly report a primary lung lesion presenting as a rare *ALK*-rearranged mesenchymal neoplasm. The patient diagnosed with primary pulmonary *ALK*-rearranged mesenchymal neoplasm (PPAMN) received ensartinib as postoperative adjuvant therapy, achieving a disease-free survival of 10 months. Continuation of ensartinib as first-line treatment enabled him to benefit from a partial response, with a progression-free survival of 11 months. Second next-generation sequencing (NGS) revealed elevated *HMBOX1::ALK* abundance along with secondary *NF2* mutation. After local radiotherapy combined with ensartinib continuation, his disease was temporarily stable for 7 months. Unfortunately, this disease became uncontrolled with an overall survival (OS) of 34 months. This is the first case of *ALK*-rearranged mesenchymal neoplasm manifested as a primary lung lesion and a novel *HMBOX1::ALK* fusion was identified by NGS. The family of *ALK*-rearranged mesenchymal neoplasms is expanding and ensartinib could be a potential treatment option for patients with *HMBOX1::ALK*. Repeated biopsy and NGS detection are critical to guide treatment selection at disease progression.

## 1 Introduction

Anaplastic lymphoma kinase (*ALK*) gene rearrangements have been progressively identified in numerous tumors (1, 2), including non-small cell lung cancer (NSCLC) (3), inflammatory myofibroblastic tumor (IMT) (4), and recently reported *ALK*-rearranged mesenchymal neoplasms (5–8). The earliest in 2016, Agaram et.al reported the first *ALK*-rearranged soft tissue tumor in a cohort of provisionally named lipofibromatosis-like neural tumors with recurrent *NTRK1* gene fusions, among which one tumor presented with *ALK* gene rearrangement replacing *NTRK* gene (9). Subsequently, mesenchymal neoplasms with *ALK* gene rearrangement were successively reported, showing spindle or eptheloid cell morphology, myxoid to myxohyaline stroma, some hyalinized vessels, occasional concentric whorls, and variable CD34 and S100 expression (5–8). Given their wide spectrum of morphology and immunophenotype, these tumors were reported with various names, such as lipofibromatosis-Like neural tumors (9), S100 and CD34 co-expressing mesenchymal neoplasms (10), infantile fibrosarcoma-like spindle cell tumors (6), and superficial *ALK*-rearranged myxoid spindle cell neoplasms (7, 11). They typically occur in cutaneous and subcutaneous tissues of trunk, limbs, head and neck. While only few cases demonstrated the origination of visceral organs, including brain (5), kidney (6), mediastinum (8), pleura (5), peritoneum (8), and bone (5, 12).

*ALK*-rearranged mesenchymal neoplasms typically present as superficial nodules or deep masses, and surgical resection with negative margins is the preferred approach. However, margin-positive cases show high rates of locoregional recurrence and metastasis, highlighting the need for other treatment strategies. In recent years, emerging *ALK* inhibitors have made significant advancements in targeted therapy for NSCLC and IMT. In 2022, crizotinib has been approved by the Food and Drug Administration in unresectable *ALK*-positive IMT patients. Ensartinib, an oral *ALK* tyrosine kinase inhibitor (TKI), exhibits superior efficacy to crizotinib in both systemic and central nervous system disease in *ALK*-positive NSCLC patients (13). Herein, we presented the first example manifested as a primary pulmonary *ALK*-rearranged mesenchymal neoplasm, who harbored a novel homeobox containing 1 (*HMBOX1*)*::ALK* fusion and sensitive to ensartinib.

## 2 Case description

A 29-year-old male, non-smoker, presented to our hospital following the identification of a mass in the left lung during a routine physical examination. Further examination revealed a mass in the left lung, measuring 14.1*10.5 cm, supposing a solitary fibrous tumor. Then, he underwent surgical resection of the mass and the left lower lobe. During intraoperative exploration, the mass was observed tightly adherent to the diaphragm and pericardium, accompanied by invasion of the pulmonary pleura.

**Figure 1:**
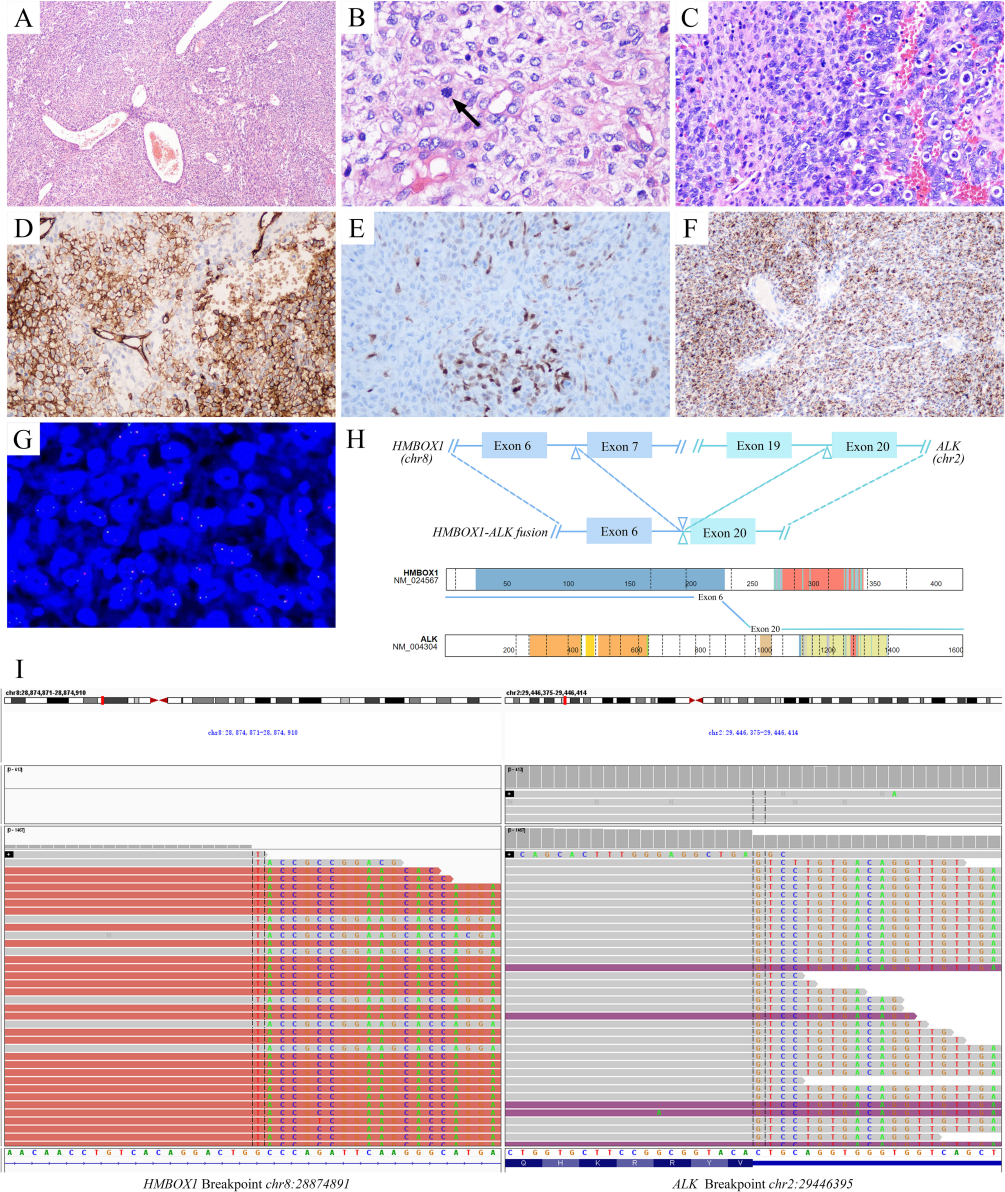
Pathological diagnosis of primary pulmonary *ALK*-rearranged mesenchymal neoplasm. Histopathologically, the tumor was composed of sheets of round, oval to epithelioid cells with background staghorn vessels **(A)**. Most of the tumor cells were mild atypia, with focal wire-like filament hyalinization around neoplasm cells. The mitosis was seen (arrow) **(B)**. In some area, the tumor cells were more cellular with increased atypia and brisk mitosis **(C)**. Immunohistochemically, the tumor cells were positive for CD34 **(D)**, S100 **(E)**, ALK-D5F3 **(F)**. More than 20% of tumor cells were signaled by break-apart *ALK* FISH assays **(G)**. Identification of *HMBOX1::ALK* fusion and schematic of predicted fusion protein encoded by it [adapted from https://proteinpaint.stjude.org/ (25)] **(H)**. IGV visualization of *HMBOX1::ALK* fusion **(I)**. *ALK*, anaplastic lymphoma kinase; FISH, fluorescent in situ hybridization; *HMBOX1*, homeobox containing 1; IGV, Itegrative Genomics Viewer.

Microscopically, the tumor was composed of sheets of round, oval to epithelioid cells with background staghorn or hemangiopericytoma-like vessels punched in (Figure 1A). Most tumor cells showed mild atypia, abundant eosinophilic to clear cytoplasm, round to polygonal nuclei, delicate chromatin, and inconspicuous nucleoli. Focally, hyalinizaed wire-like filament was presented around neoplasm cells (Figure 1B). The mitotic figures were about 1–2/10HPFs. In some areas adjoined to the pleura, the tumor cells were more cellular with increased atypia and brisk mitosis (Figure 1C). Focal hemorrhage and necrosis were also observed. Immunohistochemically, the tumor cells were positive for CD34 (Figure 1D), S100 (Figure 1E), ALK-D5F3 (Figure 1F), H3K27 me3, vimentin, and CD99 (paranuclear dot-like staining), while they were negative for STAT6, CK-pan, EMA, desmin, SMA, CD31, WT-1, and pan-TRK. The average Ki-67 index of celluar area was around 20%. Break-apart *ALK* fluorescent *in situ* hybridization (FISH) probe exhibited a positive red-green-yellow signal in over 20% of the tumor cells, suggesting *ALK* rearrangement (Figure 1G). The resected tumor tissue underwent DNA&RNA-based next-generation sequencing (NGS) (OrigiMed, Shanghai, China), revealing the fusion of *HMBOX1::ALK* (H6::A20) fusion (Figures 1H,I; Table 1). Ultimately, he was diagnosed with primary pulmonary *ALK*-rearranged mesenchymal neoplasm.

**Table 1:** Changes in NGS before and after ensartinib resistance.

Gene	Variation		Allele frequency/copy number	
			Before resistance	After resistance
*HMBOX1::ALK*	H6::A20	Fusion	20%	31%
*CDKN2A*	Deletion	CNV	0	0
*CDKN2B*	Deletion	CNV	0	0
*MTAP*	Deletion	CNV	0	0
*TERT*	c.-124C > T	SNV	35%	40%
*PTEN*	G132S exon5	SNV	38%	ND
*NF2*	G161* exon5	SNV	ND	84%

NGS, next-generation sequencing; CNV, copy number variant; SNV, single-nucleotide variant; ND, not detected.

**Figure 2:**
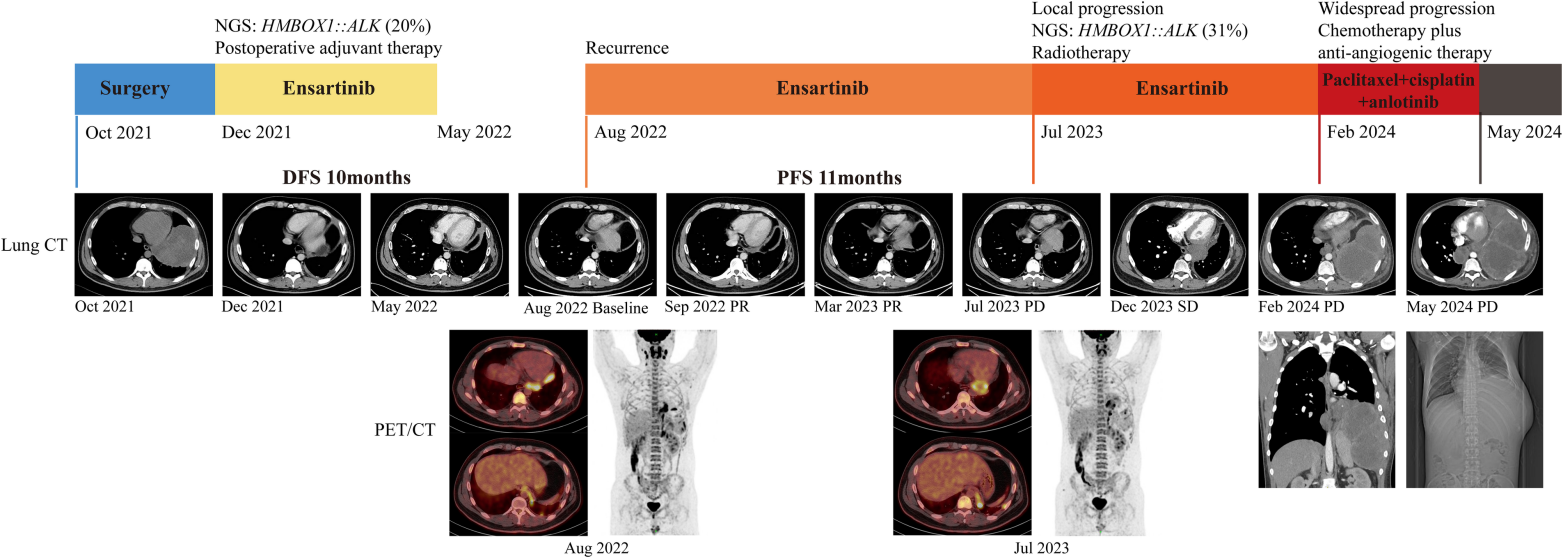
Clinical treatment history and imaging data of the patient. NGS, next-generation sequencing; CT, computed tomography; PET, positron emission tomography.

Despite remarkable advancements in *ALK* TKIs for NSCLC, their utilization as postoperative adjuvant therapy remains in clinical trials. Nevertheless, given his locally advanced stage and involvement of pulmonary pleura, he received ensartinib as postoperative adjuvant therapy based on economic affordability. Ensartinib was administered orally at a daily dosage of 225 mg from December 2021. After 5-month postoperative adjuvant targeted therapy, no recurrence was witnessed and then he discontinued ensartinib. Unfortunately, recurrence occurred 3 months later and metastatic lesions were detected in the left pleura, pericardium, and bilateral cervical lymph nodes using positron emission tomography imaging (Figure 2). Ensartinib was reintroduced as first-line therapy from August 2022. Following 1-month treatment, the enlarged lymph node sharply shrank from 4.9 × 2.6 cm to 2.9 × 1.2 cm, and partial response (PR) persisted for 11 months (Figure 2). However, a reoccurrence of mediastinal lymph node enlargement was observed along with ineffectiveness of anti-infective therapy. Regrettably, the disease progression was confirmed following a mediastinal biopsy and subsequent NGS analysis unveiled the increase of *HMBOX1::ALK* fusion (from 20 to 31% abundance) and the presence of Neurofibromin 2 (*NF2*) variant (84% abundance) (Table 1). Then he received 1-month local radiotherapy with ensartinib continuation and then a follow-up computed tomography scan revealed his disease was his disease was stable for 7 months. The patient tolerated ensartinib well, with no toxic symptoms observed during therapy. Regrettably, this disease exhibited aggressive behavior and refractoriness. Despite the administration of two cycles of chemotherapy in conjunction with anti-angiogenic therapy, his disease continued its progression unabated due to drug resistance. Subsequently, this patient was unable to tolerate anti-tumor treatment and died in August 2024.

## 3 Discussion

With the promotion of NGS the family of *ALK*-rearranged mesenchymal neoplasms is expanding. Thus far, there are around 40 cases of *ALK*-rearranged mesenchymal neoplasms and nearly 20 distinct fusion partners of *ALK* are identified among them (Table 2). Here, we provided a unique case of lung-originating *ALK*-rearranged mesenchymal neoplasm. To our knowledge, this is the first case with primary pulmonary *ALK*-rearranged mesenchymal neoplasm harboring a novel *HMBOX1::ALK* fusion and sensitive to ensartinib.

**Table 2:** Cases of *ALK*-rearranged mesenchymal neoplasms.

Year	PMID	Age	Sex	Primary Site	Fusion Partner	CD34	S100	Resection	Postoperative Therapy	Recurrence	DFS	Line of treatment	Treatment	PFS
2016	27259011	14	F	Buttock	NA	Neg	Pos	Yes		No	36 m			
2020	32222087	41	M	Intramuscularshoulder	*PPP1CB*	Pos	Pos	NA						
2020	33170538	65	M	Intraosseous vertebral	*EML4*	Pos	Pos	Yes	Radiation	No	12 m			
2021	33241586	24	F	Scalp	*EML4*	Pos	Pos	Yes		No	12 m			
2021	34464935	2	M	Hand	*ERC1*	Pos	Neg	No				First line	Crizotinib	5 m
2021	34088997	74	M	Lower back	*HMBOX1*	Pos	Pos	Yes		No	18 m			
		51	F	Upper back	*FLNA*	Pos	Pos	Yes		NA	NA			
		18	M	Lower back	*MYH10*	Pos	Pos	Yes		NA	NA			
		38	F	Thigh	*MYH10*	Pos	Neg	Yes	ALK TKI	No	6 m			
		43	M	Knee	*FLNA*	Pos	Pos	Yes		No	4 m			
		84	M	Shin	*FLNA*	Pos	Pos	Yes		No	2 m			
2022	34937902	37	F	Buttock	*FMR1*	Pos	Pos	Yes		No	9 m			
2022	35164578	9d	F	Neck	*CLTC*	Neg	Pos	No				First line	Crizotinib	NA
2022	35288525	21	F	Pelvis	*PLEKHH2*	Pos	Pos	Yes	Brigatinib	NA	NA			
2022	36387173	25	F	Thigh	*PLEKHH2*	Pos	Pos	Yes	Radiation	No	48 m			
		34	M	Neck	*EML4*	Pos	Pos	Yes		Yes	27 m			
2022	34843129	2	M	Scalp	*AK5*	Neg	Neg	Yes		No	8 m			
		2	M	Hand	*ERC1*	Neg	Neg	No				First line	Crizotinib	9 m
		2	F	Kidney	*TMP3*	Neg	Neg	Yes		Yes	1-2 m	First line	Ifosfamide+ Doxorubicin	4 m
												Second line	Ceritinib	10 m
		10	F	Kidney	*CLIP1*	Pos	Pos	Yes		No	13 m			
2023	36843055	11	M	Skin	*VCL*	Neg	Pos	Yes		NA	NA			
2023	36125853	36	F	Elbow joint	*EML4*	Pos	Pos	Yes		NA	NA			
		42	M	Pleura	*HMBOX1*	Neg	Neg	NA		NA	NA			
		59	M	Brain	*EML4*	Neg	Neg	No				NA	Radiation; Nivolumab; Alectinib	3 m
		58	F	Sternum/chest wall/sternoclavicular joint	*DCTN1*	Neg	Pos	Yes		No	72 m			
		10	M	Paratesticular mass	*PLEKHH2*	Pos	Pos	Yes		NA	NA			
		74	F	Foot	*TIMP3*	Neg	Neg	Yes		No	4 m			
		78	M	Chest skin	*FMR1*	Pos	Pos	NA		NA	NA			
		31	M	Buttock skin	*KLC1*	Pos	Neg	NA		NA	NA			
		32	M	Upper inguinal skin	*KLC1*	Pos	Neg	Yes		No	7 m			
2023	37726067	16	F	Finger	*HMBOX1*	Pos	Pos	Yes		Yes	24 m		A second resection	
		28	F	Foot	*VCL*	Neg	Neg	Yes		No	NA			
		18	F	Peritoneum/Omentum	*PRRC2B*	Neg	Neg	No				First line	Chemotherapy+ Alectinib	2 m
		11	F	Arm	*MYH10*	Pos	Neg	Yes		No	9 m			
		17	M	Shoulder	*STRN*	Pos	Neg	Yes		No	16 m			
		1 m	M	Arm	*EML4*	Neg	Pos	Yes		No	36 m			
		17	M	Mediastinum	*EML4*	Neg	Neg	No				NA	Ifosfamide+ Cisplatin+ Etoposide; Bleomycin+ Cisplatin+ Etoposide; Ifosfamide+ Methotrexate+ Cytarabine+ Etoposide+ Alectinib	NA
2024	Our case	29	M	Lung	*HMBOX1*	Pos	Pos	Yes	Ensartinib	Yes	10 m	First line	Ensartinib	11 m
												Second line	Ensartinib+ Radiation	7 m

DFS, disease-free survival; PFS, progression-free survival; F, female; M, male; NA, not available; Pos, positive; Neg, negative.

The emergence of *ALK*-rearranged mesenchymal neoplasms has brought pathologists with significant diagnostic challenges due to their diverse and distinctive morphological and immunophenotypic features. They exhibit epithelioid to round cell or spindle cell, arranged in a myxoid to hyaline or collagenous stroma, with a positive rate of 63% for CD34 and 61% for S100 (Table 2). In our case, the tumor was composed of predominantly round to epitheliod cells, with staghorn vessels and focal hyalinized stroma. Immunohistochemically, the tumor cells co-expressed CD34 and S100. Genetically, *ALK* gene rearrangement was verified by FISH and NGS. All the above evidences support the diagnosis of *ALK*-rearranged mesenchymal neoplasm. Furthermore, the tumor showed transition of low-grad area to aggressive area with increased atypia and brisk mitosis, explaining the progression of tumor and the agressiveness of biological behavior. Consistent with the findings of Gestrich et al. (8), *ALK*-rearranged epithelioid mesenchymal neoplasms in a deep location with higher-grade features follow a more aggressive clinical course.

In the clinical setting, *ALK*-rearranged mesenchymal neoplasms encompass a spectrum ranging from indolent tumors to aggressive sarcomas that are prone to local recurrence or distant metastasis. Surgical resection benefits over 70% of patients with no disease recurrence or progression (5). However, our case recurred 10 months after surgery, with metastasis involving the left pleura, pericardium, and bilateral cervical lymph nodes. Since mediastinal lymph nodes were not dissected during surgery, the potential tumor involvement remained uncertain. In addition, intrathoracic adhesions and invasion of the pulmonary pleura seem to interpretate the occurrence of metastasis to a certain extent.

*HMBOX1* gene located on chromosome 8, a member of the homeobox family (14), plays a positive role in telomere length regulation by facilitating the interaction between telomerase and telomeres (15). Degradation of *HMBOX1* mRNAs leads to progressive telomere shortening, p53 signaling inactivation, and subsequent genomic instability in cancer cells. This process drives malignancy and even progression to a more aggressive phenotype (16). To date, only three articles have mentioned the presence of *HMBOX1::ALK* fusion in *ALK*-rearranged mesenchymal neoplasms, with two cases of soft tissue (7, 8) and one case of diffuse pleural (5).

As increasing *ALK* fusions are being detected, *ALK* TKIs are revolutionizing the treatment landscape and significantly improving outcomes in *ALK*-rearranged patients (17–19). Recently, a systematic review and network meta-analysis has confirmed that ensartinib confers the most substantial progression-free survival (PFS) benefit among Asian patients with NSCLC (20). Furthermore, ensartinib is emerging as a promising therapeutic option for *ALK*-rearranged IMT, yielding favorable outcomes (21–23). Encouragingly, our case achieved a disease-free survival of 10 months, while PR was obtained with a PFS of 11 months under the administration of ensartinib.

Unfortunately, our case exhibited disease progression and subsequently acquired a mutation in *NF2*. Merlin (Moesin-ezrin-radixin-like protein) is a tumor suppressor protein encoded by *NF2* gene and plays a crucial role in tumorigenesis and metastasis (24). In our case, the glycine at position 161 of *NF2* was replaced with a stop codon, potentially impairing *NF2* function and promoting tumor proliferation. We further speculated that *NF2* mutation secondary to ensartinib may lead to drug resistance.

*ALK* TKIs offer a promising and well-tolerated treatment approach for patients with *ALK*-rearranged solid tumors, including *ALK*-rearranged mesenchymal neoplasms (Table 2). The *ALK* fusion appears to be a histology-independent target, thus future studies should consider a molecular-defined cohort approach. Given the acknowledged efficacy of *ALK* TKIs in diverse cancer types, more large-scale clinical trials are warranted to identify applicable population of *ALK* TKIs, such as patients with *ALK*-rearranged mesenchymal neoplasms. Meanwhile, it is urgent to further explore the mechanisms of resistance and explore subsequent treatment strategies.

## 4 Conclusion

This study was novel in reporting the case of a patient with primary pulmonary *ALK*-rearranged mesenchymal neoplasm harboring a *HMBOX1::ALK* (H6::A20) fusion. Additionally, we demonstrated the sensitivity of ensartinib and highlighted its potential therapeutic efficacy in *ALK*-rearranged mesenchymal neoplasms. Repeated biopsy and NGS testing are essential for guiding treatment selection, but secondary resistance remains to be addressed. We strongly believe in the immense potential of NGS-guided precision targeted therapy to deliver substantial benefits to more *ALK*-rearranged patients in the future.

## Data Availability

The datasets presented in this article are not readily available because of ethical and privacy restrictions. Requests to access the datasets should be directed to the corresponding authors.
