# Supplementary figures and images for: Ensartinib in a primary pulmonary ALK-rearranged mesenchymal neoplasm harboring a novel HMBOX1::ALK fusion: a case report and literature review
Source: Front Med (Lausanne). 2025 May 20;12:1572632. doi: 10.3389/fmed.2025.1572632 (PMC12129999; doi:10.3389/fmed.2025.1572632)

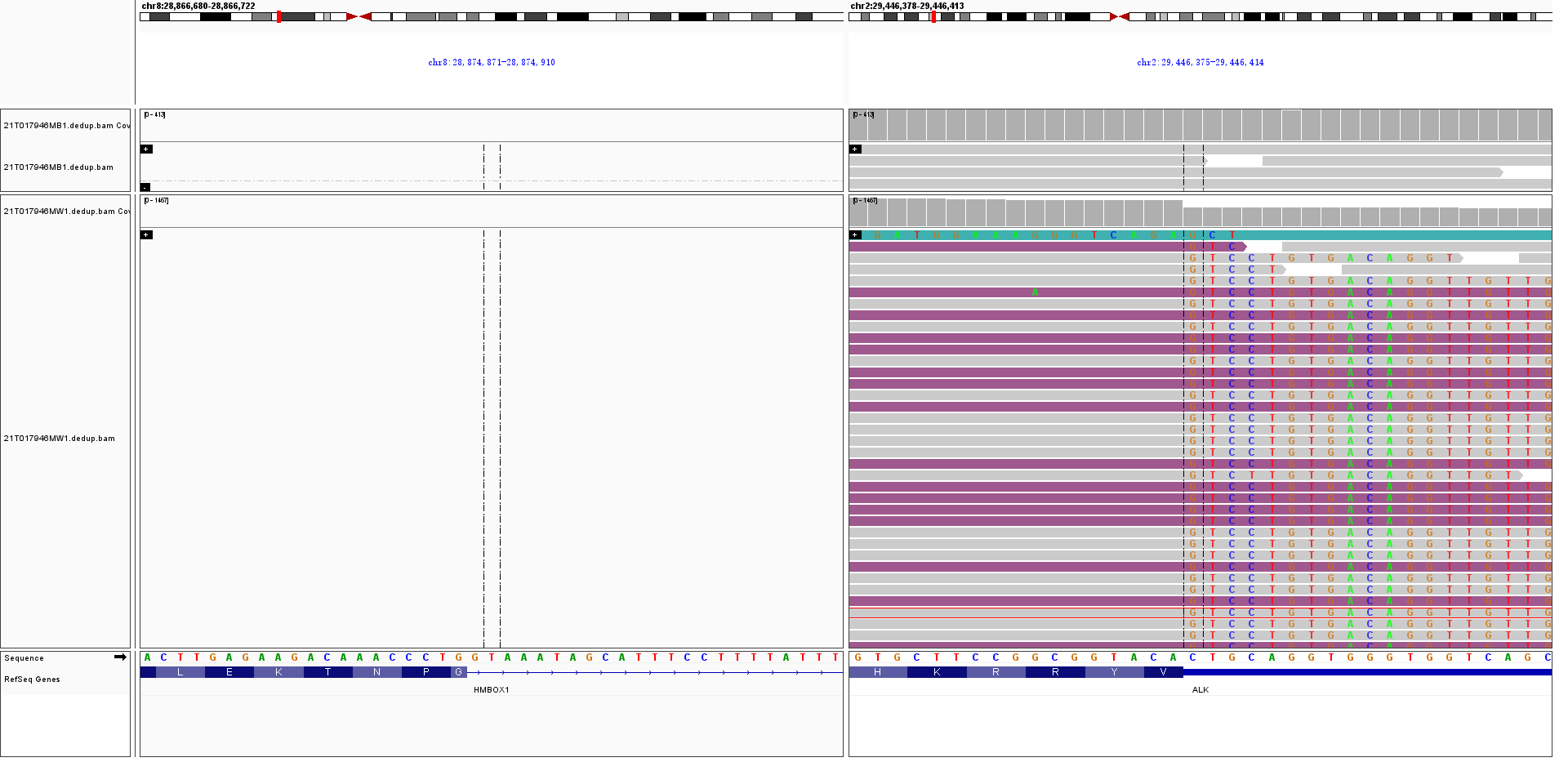

Supplement: Supplementary file 1 [file Image_1.png]
